# Supplementary figures and images for: Clinical considerations on antimicrobial resistance potential of complex microbiological samples
Source: PeerJ. 2025 Jan 28;13:e18802. doi: 10.7717/peerj.18802 (PMC11784533; doi:10.7717/peerj.18802)

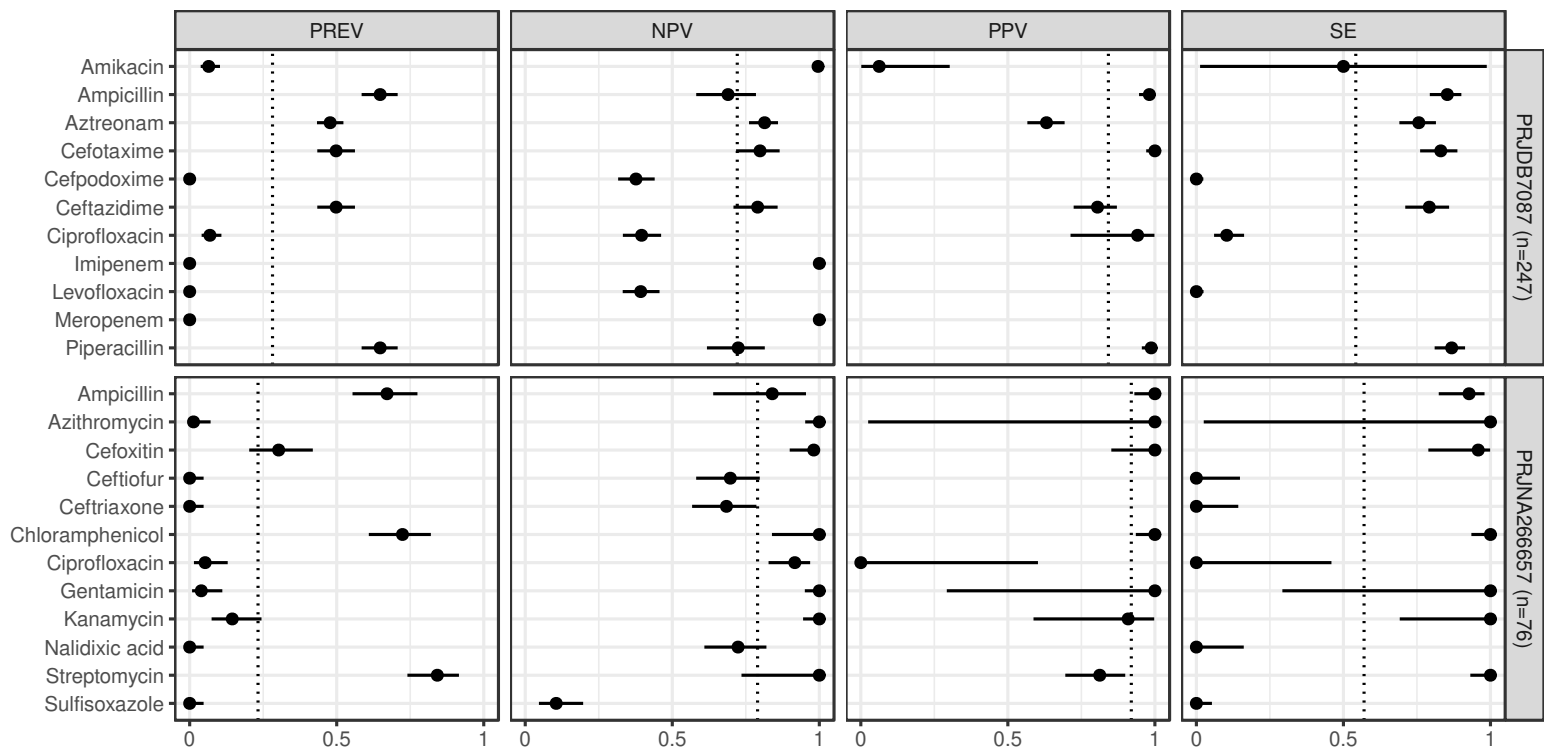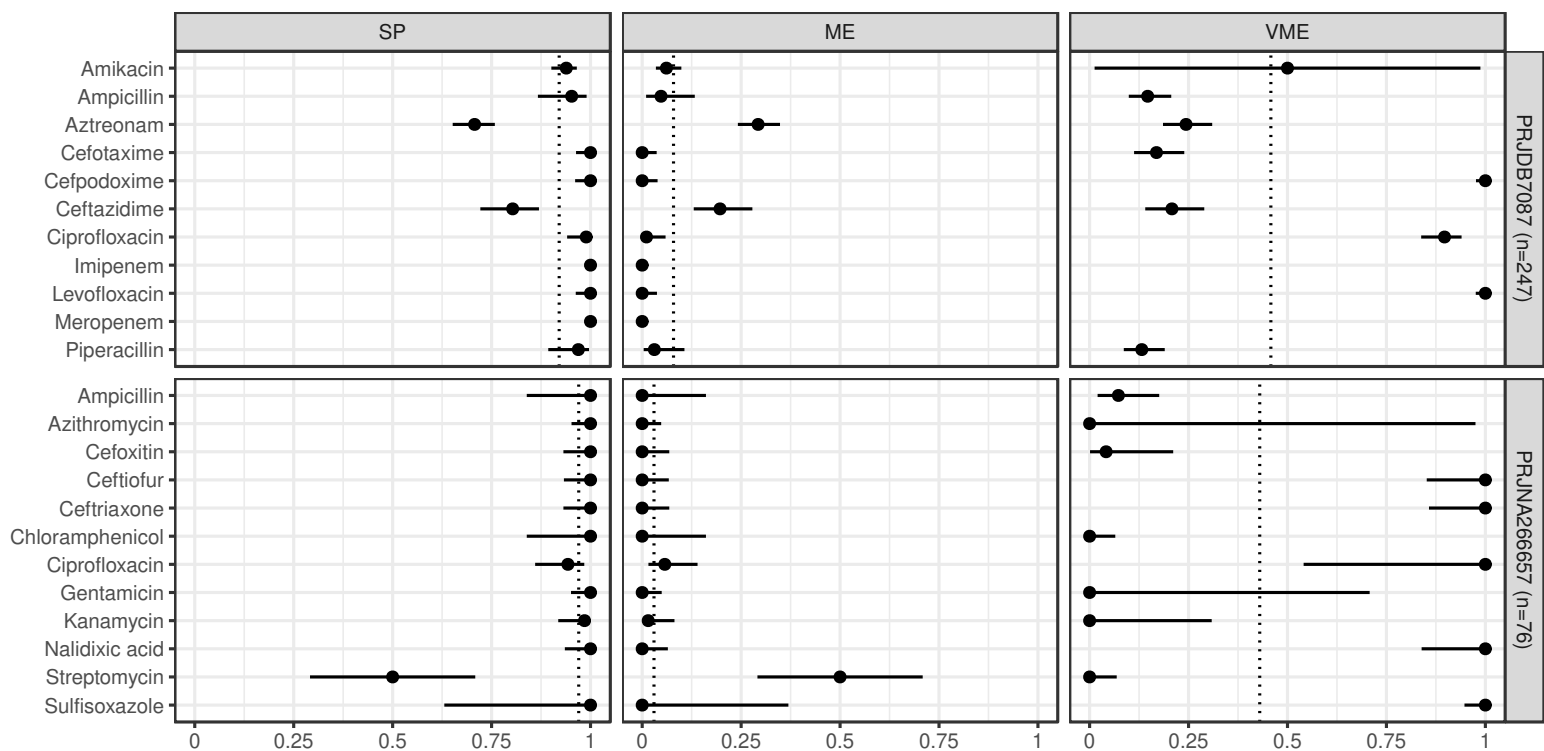

Supplement: Supplemental Information 1 — Two of the five BioProjects had intermediate AST results. In BioProject PRJDB7087 for amikacin there was one sample where the AST result was intermediate (n=1). By the rest of the ABs the number of intermediate results were as follows: aztreonam (n=61), ceftazidime (n=60), ciprofloxacin (n=6), levofloxacin (n=9), piperacillin (n=6). In BioProject PRJNA266657, intermediate results numbers were as follows: azithromycin (n=1), ceftiofur (n=5), chloramphenicol (n=1), gentamicin (n=1), nalidixic acid (n=2), streptomycin (n=19), sulfisoxazole (n=68). The calculations for the metrics assessed are based on the number of susceptibles and non-susceptibles (intermediates + resistant). Column PREV shows the prevalence (with 95% CI) of phenotypic antimicrobial resistance against certain ABs within the BioProjects. Point and 95% CI estimates for negative and positive predictive values, sensitivity, specificity, major and very major error rates are presented in columns NPV, PPV, SE, SP, ME, and VME, respectively. [file peerj-13-18802-s001.pdf]
